# Reply to Urbina, F. Are Estrogens Involved in the Earlier Onset of Psoriasis in Girls? Comment on “Cassalia et al. How Hormonal Balance Changes Lives in Women with Psoriasis. *J. Clin. Med.* 2025, *14*, 582”

**DOI:** 10.3390/jcm15010085

**Published:** 2025-12-23

**Authors:** Fortunato Cassalia, Anna Lunardon, Giovanni Frattin, Andrea Danese, Francesca Caroppo, Anna Belloni Fortina

**Affiliations:** 1 Dermatology Unit, Department of Medicine (DIMED), University of Padua, 35121 Padua, Italy; anna.lunardon@studenti.unipd.it (A.L.); giovanni.frattin@studenti.unipd.it (G.F.); francesca.caroppo@outlook.it (F.C.); anna.bellonifortina@unipd.it (A.B.F.); 2 Section of Dermatology and Venereology, Department of Medicine, University of Verona, 37126 Verona, Italy; andrea.danese_02@studenti.univr.it; 3 Pediatric Dermatology Regional Center, Department of Women’s and Children’s Health (SDB), University of Padua, 35122 Padua, Italy

We thank Dr. Francisco Urbina [1] for his thoughtful commentary on our review, How Hormonal Balance Changes Lives in Women with Psoriasis [2]. His observation on the earlier onset of psoriasis in girls adds an important epidemiologic dimension that we addressed only indirectly, and we welcome the opportunity to situate his insights within a broader mechanistic and clinical framework.

We agree that the recurring signal across cohorts—earlier onset and, in some studies, higher prevalence or greater severity in female adolescents—cannot be dismissed as incidental variation [3,4]. Even where heterogeneity in methodology, diagnostic criteria, and sampling exists, the pattern is sufficiently consistent to demand biological explanation rather than being relegated to “noise.” In our original review, we highlighted the context-dependent immunologic effects of estrogens and the clinical correlates of these effects across reproductive stages, including pregnancy, postpartum, and menopause [5,6,7]. Puberty, however, represents a particularly complex endocrine landscape: estradiol levels change rapidly, ERα/ERβ/GPER signaling is dynamic and tissue specific, and neuroendocrine axes are still maturing [8,9]. Under such conditions, hormonal influences on immunity are likely nonlinear, oscillatory, and exquisitely sensitive to environmental and genetic background, rather than following the more predictable patterns seen in pregnancy or menopause.

In this sense, we find Dr. Urbina’s emphasis on the integration of estrogen biology with other regulatory axes especially compelling. His commentary aligns well with a multidimensional model in which hormones, stress physiology, adiposity, and genetic architecture converge to shape the probability and timing of clinical disease onset.

First, the neuroendocrine stress axis is particularly relevant during puberty. This period is characterized by heightened emotional reactivity, evolving stress-coping strategies, and, in many adolescents, chronic psychosocial stress related to school, family dynamics, social media exposure, and body image. These experiences can alter cortisol rhythms, increase evening cortisol, fragment sleep, and elevate prolactin levels [10,11]. From an immunologic standpoint, such changes are not neutral: they can promote Th17/IL-23-driven inflammation and impair regulatory T cell function, potentially counterbalancing or even overriding the immunoregulatory effects that estrogens may exert in other contexts. Thus, when we consider the earlier onset of psoriasis in girls, it may be misleading to isolate estrogens as “protective” or “pathogenic.” Rather, estradiol acts within a stress-sensitive network involving corticotropin-releasing hormone, ACTH, cortisol, prolactin, and sympathetic activation.

Second, Dr. Urbina’s focus on the metabolic milieu is equally important. Puberty is accompanied by physiological increases in adiposity and transient insulin resistance, phenomena that are more pronounced in some girls due to lifestyle, diet, and socio-environmental influences. Adipose tissue is immunologically active, secreting adipokines such as leptin and resistin, as well as pro-inflammatory cytokines like TNF-α and IL-6. These mediators can potentiate Th17 polarization and enhance keratinocyte activation, thereby facilitating psoriatic inflammation [12]. Epidemiologic data suggest that higher adolescent BMI may precede the development of psoriasis in girls, and pediatric psoriasis has increasingly been associated with features of metabolic dysregulation [13,14]. In this context, estrogens interact with adipose tissue not only through classical reproductive pathways but also through modulation of adipogenesis, insulin sensitivity, and inflammatory signaling. The clinical expression of psoriasis during adolescence may therefore emerge where hormonal shifts intersect with pre-existing or evolving metabolic stress.

Third, the genetic predisposition to psoriasis remains a central pillar in this integrative model. Early-onset psoriasis is enriched for variants in HLA-C*06:02 and key components of the IL-23/Th17 pathway [14]. These genetic factors shape threshold sensitivity of immune circuits. It is plausible that sex-specific differences in receptor expression, hormonal receptor crosstalk, and epigenetic regulation confer distinct “windows of vulnerability” in girls versus boys. For instance, estrogen receptor signaling in immune cells and keratinocytes may modulate transcriptional responses downstream of IL-23 or IL-17, thereby altering the threshold at which environmental or psychological triggers manifest as clinically evident plaques. In girls carrying high-risk alleles, the convergence of pubertal hormonal flux, psychosocial stress, and increasing adiposity may be sufficient to tip the balance from subclinical inflammation to overt disease.

Within this integrative framework, estrogens are neither incidental background factors nor sole causal agents. Instead, they act as modulators within a web of endocrine, metabolic, and genetic influences. Their effects must be understood in relation to prolactin–cortisol crosstalk, adipose-derived signaling, and inherited susceptibility. This perspective helps reconcile the epidemiologic heterogeneity that Dr. Urbina highlights: differences across cohorts in stress burden, nutritional profile, genetic admixture, and age distribution may account for varying magnitudes and directions of sex differences in adolescent psoriasis. Importantly, it also suggests that simple linear models—where “more estrogen” is assumed to be uniformly protective or harmful—are unlikely to capture the true biology.

We fully endorse the call for refined research strategies, and we see Dr. Urbina’s commentary as a valuable blueprint for such work. Future pediatric and adolescent studies could be strengthened by:

Narrow age-at-onset strata and pubertal staging, using standardized clinical or hormonal criteria rather than broad chronological categories. This would help disentangle prepubertal, early-, mid-, and late-pubertal risk patterns.

Serial measurements of estradiol, progesterone, prolactin, and diurnal cortisol, ideally with high temporal resolution. Longitudinal sampling at key pubertal milestones could reveal critical transitions where hormonal shifts precede or coincide with flare onset.

Anthropometric and metabolic profiling, including BMI, waist circumference, insulin sensitivity markers, lipid profiles, and adipokine levels. This would allow formal testing of whether metabolic changes mediate or moderate the relationship between hormones and psoriasis risk.

Genetic and, where feasible, epigenetic stratification, focusing on HLA-C*06:02 status and IL-23/Th17 pathway variants, and exploring sex-specific or puberty-dependent penetrance.

Tissue-level analyses, such as receptor-signaling or cytokine profiling in lesional and non-lesional skin, are needed to define how estrogen, glucocorticoid, and prolactin receptors are expressed and activated at the local level during puberty.

Such designs would not only refine our mechanistic understanding but also enable formal mediation and interaction analyses, clarifying which pathways are most relevant to early female onset and which may be amenable to intervention.

Clinically, a synergistic model has several implications. It argues for anticipatory, personalized guidance in high-risk girls—those with a strong family history of psoriasis, rising BMI, pronounced psychosocial stress, or early metabolic abnormalities. For these adolescents, education about skin care, stress management, sleep hygiene, and weight trajectories may be particularly impactful. It also highlights the need for clinicians to recognize puberty as a critical window where early dermatologic evaluation and intervention could alter disease trajectory, potentially reducing long-term burden and comorbidities.

Furthermore, mechanistic research that dissects receptor-specific estrogen signaling in psoriatic skin, including ERα, ERβ, and GPER pathways, may ultimately inform more tailored therapeutic strategies. For example, selective modulation of estrogen signaling or crosstalk with glucocorticoid or prolactin pathways might one day complement existing biologic therapies, especially in younger female patients.

In summary, we are grateful to Dr. Urbina for stimulating this expansion of perspective. His focus on the timing and determinants of earlier onset in girls integrates well with the conceptual framework proposed in our original review, reinforcing the view that hormonal balance must be understood within a broader network of neuroendocrine, metabolic, and genetic factors. We hope that this exchange will encourage future age-specific studies that can follow patients from childhood through reproductive age and into menopause. This could help translate evidence into timely and life-stage-specific management of women with psoriasis [2].
